# Breast cancer inter-image dissimilarity by feature optimization: An application of novel flea optimization algorithm

**DOI:** 10.1371/journal.pone.0341848

**Published:** 2026-02-09

**Authors:** P.P. Fathimathul Rajeena, Muhammad Yasir, Mona. A. S. Ali, Junaid Ali Khan

**Affiliations:** 1 Computer Science Department, College of Computer Science and Information Technology, King Faisal University, Alhasa, Saudi Arabia; 2 Department of Computer Science, HITEC University Taxila, Taxila, Pakistan; 3 Department of Computer Engineering, Sakarya University, Serdivan/Sakarya, Türkiye; National University of Science and Technology POLITECHNICA Bucharest, ROMANIA

## Abstract

**Background/Objective:** Breast cancer is a serious disease that has caused thousands of deaths around the world. According to the American Cancer Society, more than 40,000 women and about 600 men lost their lives due to breast cancer in 2021, and it increased to 43,700 women and 530 men until 2023.

**Method**: In this paper, a modified version of ResNet-50 has been exploited to extract features from breast tissue biopsy slides, contained in the BreakHis public dataset. The standard 177 layer model is amended upto 146 layers by reducing redundant activation, normalization operations and number of convolutional filters without compromising representational capacity. As a result the computational efficiency is achieved along with reduction in learnable parameters from 23.7M to 16.8M. The features vector is extracted using novel Flea optimization Algorithm that performs exploration from a d-dimensional search space to get global features. An inter-image dissimilarity evaluation has been performed to find out class compactness and separation, demonstrating its crucial role in achieving better classification performance. The results of the proposed framework are obtained on various performance indicators including average accuracy, precision, recall, F1 score etc while the statistical analysis is made to see the reliability of the framework based on MCC, Cohen’s Kappa and t-test.

**Results:** The results of the proposed method are compared with DenseNet, VGG, CNN with LSTM, Primal Dual Multi-instance SVM, Single Task CNN and Multi Task CNN and shown dominance on various performance measures. An accuracy of 99.20% was achieved at 40× magnification, 99.62% at 100× magnification, 99.50% at 200× magnification, 99.34% at 400× magnification, respectively.

**Conclusions:** The proposed approach, implemented on the real hardware, can provide an alternate to health experts in diagnosing breast cancer in the early stages.

## Section I: Introduction and related literature

Breast cancer is a serious disease that has caused thousands of deaths around the world. According to the American Cancer Society, over 40,000 women and about 600 men lost their lives due to breast cancer in 2012 in USA and the count increased to 43,700 women and 530 men till 2023. Whereas, breast cancer has resulted 6,85,000 deaths all over the world during 2023 [1–3]. There are four main types of breast cancer: benign, normal, in situ carcinoma, and invasive carcinoma. A benign tumour changes the breast slightly, but it is not harmful or dangerous. In situ carcinoma only affects certain parts of the breast and doesn’t spread to other body parts [4]. It is not very harmful and can be treated if found early. The most dangerous type is invasive carcinoma, which can spread to other organs [5–7]. Healthcare professionals can detect breast cancer using methods like mammograms, X-rays, Portion Emission Tomography (PET), Ultrasound, Magnetic Resonance Imaging (MRI) and Computed Tomography (CT). if detected at initial stages, the cancer can be cured [8]. The best way to diagnose breast cancer is by looking at tissue from the breast under a microscope. To make the tissue easier to see, it is stained with special dyes in a lab. Histopathological image analysis (which looks at microscopic images of breast tissue) is important for early cancer treatment. Genomics is the study of genes, also helps in understanding breast cancer. By combining data from genes and medical images, doctors can make more accurate diagnoses [9,10]. Computer-Aided Design (CAD) has been used to help identify breast cancer. Whereas, traditional CAD systems rely on features created by people, which can reduce their effectiveness. With new advances in technology, deep learning methods have been used to detect breast cancer. Deep learning is better at solving complex problems because it doesn’t need as much human involvement [11,12]. This makes it useful for things like natural language processing, image analysis, pattern recognition [13,14]. Moreover, ML has played an important role in the field of medical imaging especially, in feature extraction and transfer learning [15].

In [16], the authors have performed classification on BreakHis dataset. In this approach, experiments are performed by using different magnification levels of histopathological images and for classification, Support Vector Machines (SVM), 1-Nearest Neighbour (1-NN), Random Forest (RF), and Quadratic Linear Analysis (QDA) are utilized. After experiments, maximum 85% accuracy has been achieved. The authors have highlighted the issues related to the dataset such as multiclass classification which is a hard task to classify. Although their work has laid the basis for new dataset in cancer research but, their proposed method has many shortcomings including high false positive rate and curse of dimensionality.

S. H. Kassani et al. [17] have proposed an ensemble approach which used three pretrained deep convolutional neural network (CNN) models for feature extraction. At preprocessing stage, augmentation, stain-normalization, tuning of hyper-parameters has been performed. Moreover, fine-tuned convolutional neural networks model has been used in the study to achieve improved results. These models include VGG19, DenseNet and MobileNet. The extracted features are fed to multi-layer perceptron for classification. For validation of proposed model, BreakHis, Patch Camelyon, ICIAR, and Bioimaging datasets were used. The study achieved 98.13%, 95%,94.6% and 83.10% accuracies respectively. The combination of different deep neural network approaches not only increased computational complexity but it also increased the time complexity of the proposed approach.

By utilizing the BreakHis dataset, I. Sayin et al. have used Xception, VGG, InceptionResNet and ResNet deep learning pre-trained models for classification of histopathological images of breasts. They came to conclusion that Xception outperformed all models in comparison with the accuracy of 89% whereas, InceptionResNet and ResNet gave 87% accuracy but their F-1 Score was different; former gave 86 and later provided 87. The authors only utilized the 200× magnified data of the images, they have not tested their proposed approach on other magnification levels of the dataset. Likewise, they have obtained low classification accuracy by using their proposed approach [18].

A. Nahid et al. have exploited the statistical and structural information of BreakHis dataset images for classification of histopathological images using CNN, Long-short-term Memory (LSTM) and combination of CNN & LSTM. First extraction of the features is performed by using aforementioned models then Support Vector Machine (SVM) with Softmax function is used for final classification. They obtained maximum 91% accuracy at 200× magnification level and maximum precision was 96% at 40× magnification level whereas maximum F-1 measure was observed at 40× & 100× magnification levels. The authors have not evaluated all the magnification level as a whole but they have processed turn by turn all magnification levels which does not generalizes the whole dataset [19]. To detect the malignant images in BreakHis dataset, a Primal-Dual Multi-Instance SVM was introduced by H. Seo et al. they also derived an optimization algorithm for SVM without using quadratic programming and least-squares. The authors claimed that this reduces the complexity of the optimization and increases the scalability of the algorithm. Although, the authors have computed the accuracy on all magnification levels of BreakHis dataset but they achieved maximum 89.8% accuracy at 200× magnification level with 92.4 and 63.6 F-1 score and recall respectively [20].

In their monograph, K. Jabeen et al. have presented a pre-processing image enhancement technique and named it haze-reduced local-global which improves the contrast of the images. Later on, they performed augmentation on the dataset and fed the pre-processed dataset to the pre-trained EfficientNet-bO model. They extracted the features from pre-trained model and fed them to the optimization algorithm for feature selection using Equilibrium-Jaya controlled Regula Falsi. The authors have used two datasets in their study; i) INbreast & ii) CBIS-DDSM. They achieved 95.4% and 99.7% accuracy on both datasets respectively. The authors have used smaller datasets and by using comparatively a larger dataset like BreakHis one may be able to check the effectiveness of their proposed approach [21]. In [22] D. Kaplun et al. have used Zernike image moments for extraction of features from histopathological images and used neural networks to classify the extracted features. The authors also utilized the Explainable Artificial Intelligence and Local Interpretable Model-Agnostic Explanation techniques to explain their model. They have claimed that they have achieved 100% accuracy at 40× magnification while performing binary classification. Their proposed model needs meticulous review as their classification accuracy is claimed too high. Moreover, use of interpretation methods have not only increased the computational complexity rather, proposed approach has increased the time complexity as well.

In [23], the authors have performed classification on BreakHis dataset independent from magnification levels of images by using CNNs. They have used two models: single task CNN for classification of malignant images and multi-task CNN for prediction of benign and malignant images. They have stated that their approach has performed well for classification. Moreover, they have also claimed that their proposed model can capture additional information from different magnification levels. They achieved maximum 83.33% accuracy from their proposed model. They have not utilized the whole BreakHis dataset. Instead, they have used a subset of the dataset for their research. Use of complete dataset may affect the performance metric. Table 1 represents the comparison of state of the art approaches.

**Table 1:** Comparison of state of the art approaches.

Related work	Methodology	Accuracy (%)	Dataset Used	Short coming
S. H. Kassani et al. [17]	VGG19, DenseNet and MobileNet	98.13%	BreakHis, Patch Camelyon, ICIAR	Increased time and computational complexity
I.Sayin et al. [18]	Xception, VGG, InceptionResNet and ResNet	89%	BreakHis	Low accuracy, only 200× magnification level was used for study
A. Nahid et al. [19]	CNN & LSTM	91%	BreakHis	Low accuracy only 91%
H. Seo et al. [20]	Primal-Dual Multi-Instance SVM, Quadratic Programming	89.8%	BreakHis	Low accuracy onyl 89.8%
K. Jabeen et al. [21]	EfficientNet-bO, Equilibrium-Jaya controlled Regula Falsi	95.4% & 99.7%	INbreast & CBIS-DDSM	Smaller datasets (410 and 3100 instances respectively) were used in the study.
N Bayramoglu et al. [23]	Single task CNN, multi-task CNN	83.33%	BreakHis	Low accuracy, A subset of whole dataset was used in the study.

The contributions of this work are as follows:

- Proposed modified ResNet-50 based deep neural network architecture having 116 convolution layers consisting of 35 convolution 35 batch normalization 01 max pooling 01 average pooling 31 activation 10 addition 01 softmax 01 input and 01 output layers.
- A novel Flea Optimization Algorithm is presented for selection of optimized set of features to perform effective classification.
- The results of the proposed approach have been compared with VGG, DenseNet, ResNet-50, CNNs, InceptionResNet, and LSTM.
- An ablation study has been conducted to find the effectiveness of proposed approach.
- Simulations have been performed for 1000 number of iterations to check the effectiveness of proposed approach by meticulously observing the time, space and computational complexity in addition to convergence and accuracy.

The rest of the paper consists of four sections. The general understanding of the model, Flea Optimization Algorithm, the performance measures, revised ResNet-50 model, and feature optimization are described in Section II, under the proposed work. The values of the parameters of simulation environment are stated in section III along with the explanation and the discussion of the results. In section IV, after completing the subject studies, we have provided conclusions, recommendation for the subsequent research and direction for the said study.

## Section II: Proposed work

The section breaks down into four sub-units. The modified ResNet-50 Model is explained in the first section, in the second section, Flea Optimization Algorithm is described, and the feature optimization technique is explained in the last section.

### Modified ResNet-50 model

**Fig 1:**
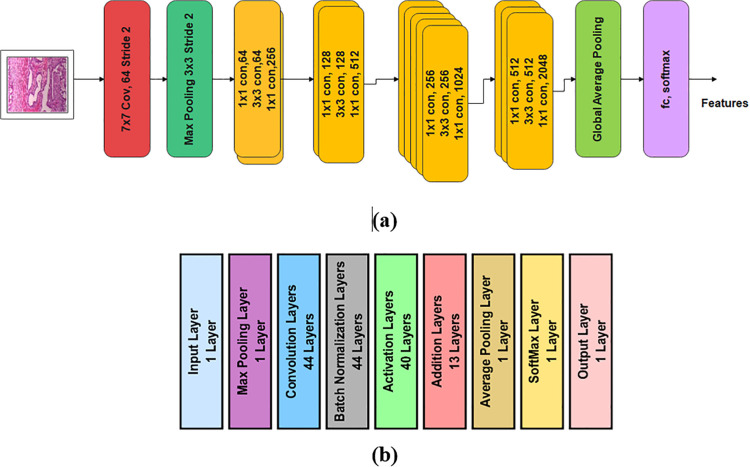
Modified ResNet-50 model: (a) Architecture, (b) block diagram.

The modification in ResNet50 model by reducing the number of learnable parameters and layers, results optimal feature selection which consequently helps in better classification of images. Moreover, it also consequent reduction in training time and improvement in prediction accuracy. In this modification, the total number of layers are 146 and the number of learnable parameters decreased from 23.7M to 16.8M, thereby reducing the model cost. These 146 include: 01 input 44 convolution 44 batch normalization 01 max pooling 01 average pooling 40 activation 13 addition 01 SoftMax and 01 output layer. The layered architecture along with block diagram of modified ResNet50 is shown in Fig 1.

The Table 2 presents the number of learnable parameters and layers for standard ResNet-50 and modified ResNet-50, is given below for comparison.

**Table 2:** ResNet-50 and modified ResNet-50 comparison.

Parameter Name	ResNet-50	Modified ResNet-50
Input layers	1	1
Convolution layers	53	44
Batch Normalization Layers	53	44
Max Pooling Layers	1	1
Average Pooling Layers	1	1
Activation Layers	49	40
Addition Layers	16	13
Softmax Layers	1	1
Output layers	1	1
Classification Layer	1	0
Total number of layers	177	146
Number of learnable parameters (in Million)	25.6	16.8

The output of trained network is used to train deep neural network using transfer learning which provides a feature vector at output. To obtain discriminant features and reduce the dimensionality of feature vector, feature optimization is performed by using Flea Optimization Algorithm. Later on, it is fed to different classifiers to find inter-image dissimilarity.

### Flea optimization algorithm

**Fig 2:**
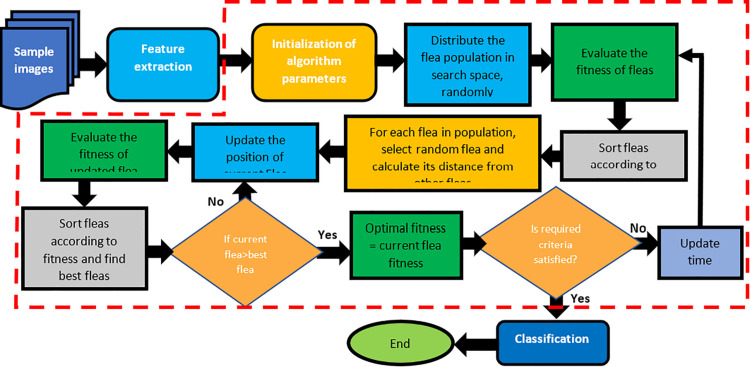
Work flow of proposed methodology.

A model to select a subset of discriminating features from a given feature vector has been presented in this monograph. The objective of the optimization is to achieve an optimized feature set that may accurately classify the instances to improve the performance metric. This study has introduced a novel Flea Optimization Algorithm for selection of optimal features from another feature set, obtained using modified ResNet50 model, as explained earlier in section II-A. The proposed approach follows following key steps: (i) image pre-processing, (ii) feature extraction, (iii) feature optimization, and (iv) classification of images as per their discriminant features. Flea Optimization Algorithm (FOA) is used as a meta-heuristic search algorithm that optimizes the subset of ResNet-extracted features. Unlike standard LASSO solvers, which only perform convex coefficient shrinkage on all input features, FOA explicitly searches for an optimal combination of relevant features by encoding feature-selection masks within the flea population. Standard LASSO solver optimize coefficients under L1 penalty but do not explore combinations of features beyond convex shrinkage. Therefore, LASSO may retain correlated features but it may exhibit sensitivity to the regularization parameter and may fail to identify the global optimum in high-dimensional feature spaces. In contrast, FOA performs a population-based global search that explicitly evaluates feature-selection vectors. This allows FOA to discover more discriminative subsets of the ResNet features than LASSO alone, especially when the data distribution or extracted features are non-linear and multi-modal. This bi-level optimization enables FOA to remove redundant or noisy ResNet features, avoid the limitations of purely convex sparsity penalties, and escape local minima when the feature space is high-dimensional. Graphical representation of proposed models is shown in Fig 2.

At start, pre-processing steps is performed on input images by augmentation technique to enhance the model’s generalization and robustness. To achieve this, we have applied:

- A random rotation ranging from –5 to 5 degrees
- Random X-axis and Y-axis reflection (to introduce variability to the training images).
- Random X-axis and Y-axis shear within the range from –0.05 to 0.05 (To address geometric distortions in the images)
- At last, images are resized to 224×224 before feeding to the modified ResNet50.

At modified ResNet-50 stage, the preprocessed input images are divided into 70/30 training and validation instances then feature extraction process is performed. We have tried different ratios of the dataset for training and testing like 80/20, 60/40 but 80/20 provided biasness and 60/40 was not able to converge the model in correct direction. 70/30 avoids both aforementioned problems. The output of trained network is used to train deep neural network using transfer learning. The output of modified ResNet-50 is a Directed Acyclic Graph (DAG) which is used for feature extraction from BreakHis dataset.

The modified ResNet50 model’s output serves as the input for the Flea Optimization Algorithm, which conducts optimizations based on fitness criteria. Features with higher fitness values are prioritized in this process. The features are index-sorted in descending order, placing those with greater fitness values at the beginning indices. Then Least Absolute Shrinkage and Selection Operator (LASSO) objective function is used with FOA to reduce the dimensions of feature vector. Subsequently, the optimized set of features is fed into the classifier to find inter-image-dissimilarity. Our study includes Decision Trees, Narrow Neural Network, Medium Neural Network, Wide Neural Network, Bi-layer Neural Network, and Tri-Layers Neural Network to find inter-image dissimilarity.

The population of ‘s’ fleas with ‘t’ parameters of each flea which is equal to input feature vector as:

$$F=(\begin{array}{ccccc} f_{1,1} & f_{1,2} & f_{1,3} & \cdots & f_{1,t}\\ f_{2,1} & f_{2,2} & f_{2,3} & \cdots & f_{2,t}\\ \vdots & \vdots & \vdots & \ddots & \vdots \\ f_{n,1} & f_{n,2} & f_{n,3} & \cdots & f_{n,t}\\ \vdots & \vdots & \vdots & \ddots & \vdots \\ f_{s,1} & f_{s,2} & f_{s,3} & \cdots & f_{s,t} \end{array})$$ (1)

The feature vector for the *i*–*th* instance is denoted as $L_{i}=\{f_{i,1},f_{i,2},f_{i,3},\cdots ,f_{i,t}\}$. To achieve our optimization objective, we employed the least absolute shrinkage and selection operator (LASSO). LASSO modifies the ordinary least squares (OLS) objective function by incorporating a penalty term. This penalty term encourages the selection of a smaller, more relevant subset of features by driving some of the coefficients to zero. This process simplifies the model and enhances its predictive accuracy. The mathematical expression for LASSO is written as:

$$J(\psi )=\arg\min_{\psi }\left(\frac{1}{2N}\|f-L\psi {\|}^{2}+\alpha \|\psi {\|}_{1}\right)$$ (2)

In above equation, *f* denotes the actual outcome, *L* is the feature matrix, *α* is the regularization parameter that defines the strength of the shrinkage term, and *ψ* is the vector of coefficients, we intend to estimate. Increasing the value of *α* results in greater shrinkage of the coefficients, which means more coefficients are reduced to zero. Above equation comprises two components: ‖f − $L\psi {\|}^{2}$ represents the ordinary least squares term, specifically the squared Euclidean norm (L2 norm) of the residual sum of squares. It indicates how well the model is performing on the training data; and $\|\psi {\|}_{1}$ denotes the L1 norm of the coefficient vector, which sums the absolute values of the coefficients. The L1 norm serves as a penalty term, causing some coefficients to become zero depending on the value of *α*. Coefficients that are zero are excluded, leaving the remaining features selected for classification. The objective is to find the values of *ψ* that minimize this objective function. The logical steps of FOA are given in Algorithm 1.

**Algorithm 1. Algorithm-1: Flea optimization algorithm for feature selection.**



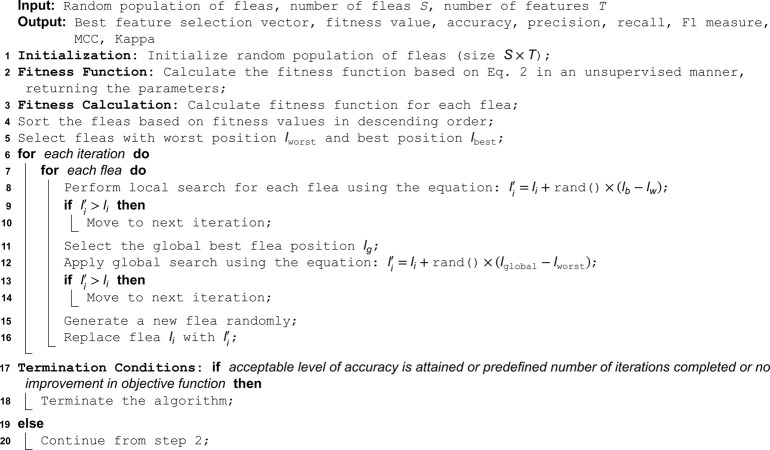



### Feature optimization

In feature optimization, LASSO is used as objective function which uses convex penalty function to reduce the number of features, as described in the previous section (B). When the value of *α* is larger, more features are set to zero, leading to a smaller set of features being selected for classification. Conversely, a smaller value of *α* results in fewer features being set to zero, which means more features are retained for classification. This may negatively impact classification accuracy and time complexity. On the other hand, fitness of the discriminatory features is selected by FOA. It performs exploration and exploitation is search space and selects the discriminatory features by utilizing the intelligence of its population. FOA selects optimal features by avoiding local optima in d-dimensional space whereas LASSO reduces the number of selected features so that most optimal feature set may be obtained. Experiments with various values of *α* have been performed and it was found that *α* at value of 0.0030 strikes the right balance. It significantly reduces the number of features while it also helps to maximize classification accuracy. Jointly, FOA and LASSO reduced number of features from 2048 to 780, 811, 664, 733 for 40×, 100×, 200× and 400× magnification levels, respectively. This reduction decreased computational time and storage costs while improving accuracy and convergence.

## Section III: Results and analysis

This section enlists the key findings which have been observed during the study. To carry out the study, we have used MatLab R2022a for simulations on Intel Core i5 8350U CPU in Windows11 environment. Tree, Narrow Neural Network, Medium Neural Network, Wide Neural Network, Bi-Layered Neural Network and Tri-Layered Neural Network have been used for classification of features. Table 3 shows the general and specific parameter values and settings, used in the experiments:

**Table 3:** Hyperparameters, their values and settings.

Training Parameters	
Parameter	Values/Settings
Number of Features	2048
Number of Folds	10
Rotation in Images	$-5\leq x\leq 5^{\circ }$
Random Shear in Images	$-0.05^{\circ }\leq x\leq 0.05^{\circ }$
Learning Rate during Training	0.0002
Mini Batch Size	20
Activation Function for Training	Soft Max
Other settings	Default
**Optimization Parameters**	
Learning Rate during Optimization	0.0001
Resilin	Rand (0 -1)
Flea Population	20
Max iterations	690,720,700,630
*α* for LASSO	0.0030
Other settings	Default

Performance metric for evaluation of proposed approach along with mathematical equations is given in Table 4.

**Table 4:** Performance measures and their equations.

Measure	Equation
Accuracy	$\frac{TP+TN}{TP+TN+FP+FN}$
Error	$\frac{FP+FN}{TP+TN+FP+FN}$, or 1−Accuracy
Sensitivity	$\frac{TP}{TP+FN}$
Specificity	$\frac{TN}{TN+FP}$
False Positive Rate	$\frac{FP}{FP+TN}$
F1 Score	$\frac{2PR}{P+R}$
MCC	$\frac{\left(TP\cdot TN)-(FP\cdot FN\right)}{\sqrt{\left(TP+FP)(TP+FN)(TN+FP)(TN+FN\right)}}$
Cohen’s Kappa	$\frac{P_{o}-P_{e}}{1-P_{e}}$
**Where:**	
$P_{o}=\frac{TP+TN}{TP+TN+FP+FN}$	
$P_{e}=\frac{\left(TP+FP)(TP+FN)+(FN+TN)(FP+TN\right)}{(TP+TN+FP+FN){\;}^{2}}$	

**Fig 3:**
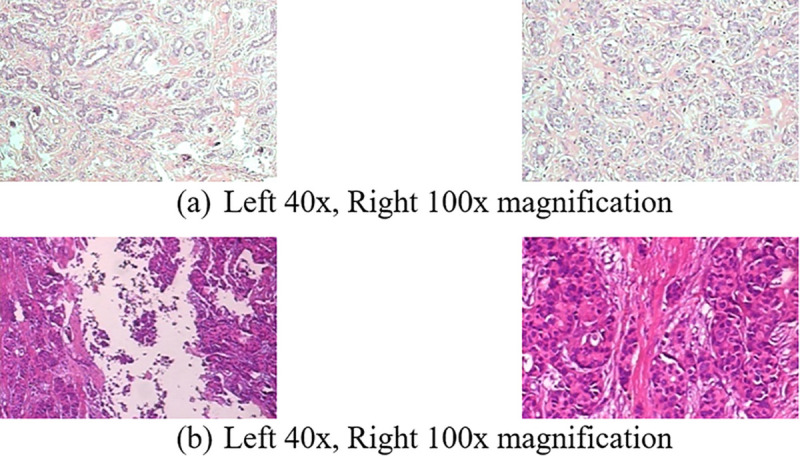
Breast cancer images of dataset (a) adenosis (b) ductal carcinoma.

BreakHis dataset have been used in our study, which consists of histopathological images of breasts cancer patients. It has 7909 microscopic images of breast cancer tissues with two classifications: benign and malignant. To capture the dataset, 40×, 100×, 200× and 400× magnification levels has been used. The original dataset has two main classes: benign and malignant then each class has its associated cancer sub-class, the cancer subclasses have four types of magnification levels for breast tissues images i.e. 40×, 100×, 200× and 400×. It has eight classes: four for malignant type: Ductal Carcinoma (DC), Lobular Carcinoma (LC), Mucinous Carcinoma (MC), Papillary Carcinoma (PC) and four for benign type: Adenosis (A), Fibroadenoma (F), Phyllodes Tumor (PT), Tubular Adenoma (TA) [24]. Fig 3 shows the sample images of the dataset from 40× and 100× magnification level.

The dataset images have been classified as per their inter-image dissimilarity. Five standard MATLAB neural-network classifiers were used for classification, including Narrow, Medium, Wide, Bi-Layered, and Tri-Layered neural networks. These models differ in depth and number of neurons. The Narrow, Medium, and Wide models contain a single hidden layer with approximately 10, 20, and 50 neurons, respectively, whereas the Bi-Layered and Tri-Layered models include two and three hidden layers with 10 neurons per layer. All networks were trained using MATLAB’s default scaled Conjugate Gradient Training (SCG) algorithm, TanSig activation function, softmax output layer, default data division and stopping criteria. Training was performed using Stochastic Gradient Descent with Momentum (SGDM) to train the deep learning model. The dataset was divided into 70/30 training and validation subsets using stratified splitting. To improve generalization, data augmentation was applied, including random rotation (±5^°^), horizontal/vertical reflection, and random shear (±0.05). All images were resized to 224×224×3 using grayscale-to-RGB conversion where needed.

The modified ResNet architecture was fine-tuned by replacing the softmax, classification and final fully connected, layers with new softmax, classification and fully connected layers. Final layer has eight neurons to accommodate the classification of the dataset into eight classes. Hyperparameters used for training include: Learning Rate 0.0002, Batch Size 20 with 10 number of epochs. Moreover, shuffling was performed in every epoch and validation was carried out by using augmented validation set. Experiment results have shown significant increase in accuracy, precision and recall in addition to improvement in MCC, F-1 measure and Kappa. Moreover, error and false positive rate has decreased, as shown in graphs in this section, later. Table 5 depicts the performance metric before feature optimization whereas Table 6 display the classification results after feature optimization. It can be observed from the Tables 5 and 6 that proposed approach has outperformed in all performance measures after feature optimization.

**Table 5:** Classification results at 40× magnification, before feature optimization on BreakHis dataset. Boldface values depict significant results.

Performance Metric	Classifier					
	Tree	Narrow NN	Medium NN	Wide NN	Bi-Layer NN	Tri-Layer NN
**Accuracy**	0.5709	0.8623	0.9015	**0.9065**	0.8342	0.8233
**Error**	0.4291	0.1377	0.0985	**0.0935**	0.1658	0.1767
**Recall**	0.4537	0.8422	0.8769	**0.8845**	0.7926	0.7736
**Specificity**	0.9315	0.9784	0.9838	**0.9843**	0.9739	0.9726
**Precision**	0.4635	0.8292	0.8932	**0.8972**	0.7983	0.7738
**False Positive Rate**	0.0685	0.0216	0.0162	**0.0157**	0.0261	0.0274
**F1_Score**	0.4575	0.8348	0.884	**0.8899**	0.7943	0.7729
**MCC**	0.39	0.8134	0.8685	**0.8751**	0.7687	0.7458
**Kappa**	0.4903	0.3706	0.5497	**0.5727**	0.2419	0.1922
**Training Time(Sec)**	**17.271**	21.798	25.701	48.265	89.581	113.8

**Table 6:** Classification results at 40× magnification, after feature optimization on BreakHis dataset. Boldface values depict significant results.

Performance Metric	Classifier					
	Tree	Narrow NN	Medium NN	Wide NN	Bi-Layer NN	Tri-Layer NN
**Accuracy**	0.9879	0.9899	0.9899	**0.992**	0.9709	**0.992**
**Error**	0.0121	0.0101	0.0101	**0.008**	0.0291	**0.008**
**Recall**	0.9841	0.9848	0.9846	0.9878	0.9538	**0.9884**
**Specificity**	0.9982	0.9985	0.9985	**0.9988**	0.9958	0.9987
**Precision**	0.9816	0.9863	0.9864	**0.987**	0.9619	0.9886
**False Positive Rate**	0.0018	0.0015	0.0015	0.0012	0.0042	**0.0013**
**F1_Score**	0.9828	0.9854	0.9855	0.9873	0.957	**0.9885**
**MCC**	0.981	0.984	0.984	0.9862	0.9534	**0.9873**
**Kappa**	0.9449	0.9541	0.9541	0.9632	0.8668	**0.9633**
**Training Time(Sec)**	**6.2643**	12.808	16.443	15.246	13.031	22.535

It is evident from Table 5 that maximum accuracy achieved, before feature optimization is approximately 90% which is attained by using Wide NNs. Similarly, maximum precision is 89.72% whereas recall is 88.45% which is also exhibited by Wide NN. After feature optimization, maximum accuracy is 99.2% which is obtained by using Wide NNs and Tri-Layer NNs. Likewise, both recall and precision increased to 98.84% and 98.78% respectively in case of Tri-layered NN and Wide NNs. All other classification algorithms performed with more than 97% accuracy. It is worth mentioning that false positive rate has been reduced from 0.0157 to 0.0012 which testifies the effectiveness of the proposed approach. Moreover, the training time also reduced after reduction in number of features. For example, it took Trilayered NN to train 113 seconds before feature optimization and after feature optimization, it only took 22.535 seconds. In the same way, training time for all classifiers has reduced after feature optimization. Due to comparatively simple internal structure of the decision trees, they took less time as compared to neural networks, which have comparatively complex internal architecture.

For 100× magnification level, Tables 7 and 8 represent the classification results before and after optimization, respectively. In this case, before optimization, the maximum accuracy attained is 89.70% with 89.83% and 83.68% precision and recall respectively. The error was 10.30% with 1.75% false positive rate. After feature optimization, Trees outperformed all classifiers, maximum accuracy achieved is 99.62% with 99.58% precision and 99.29% recall whereas, the error and false positive rate have been reduced to 0.38% and 0.061% respectively. The reduction in features not only resulted decreased training time but it also resulted a decrease in the complexity of inputs as a result, Trees performed better. Hence these graphs represents the effectiveness of proposed approach. In case of neural networks, the narrow, medium, wide and bilayered NN provided more than 99% accuracy.

**Table 7:** Classification results at 100× magnification, before feature optimization on BreakHis dataset.

Performance Metric	Classifier					
	Tree	Narrow NN	Medium NN	Wide NN	Bi-Layer NN	Tri-Layer NN
**Accuracy**	0.5457	0.8662	0.8739	**0.897**	0.8296	0.8056
**Error**	0.4543	0.1338	0.1261	**0.103**	0.1704	0.1944
**Recall**	0.4659	0.8337	0.8492	**0.8668**	0.8017	0.7535
**Specificity**	0.9271	0.9782	0.9791	**0.9825**	0.9737	0.9699
**Precision**	0.4579	0.8398	0.8648	**0.8983**	0.7866	0.7563
**False Positive Rate**	0.0729	0.0218	0.0209	**0.0175**	0.0263	0.0301
**F1_Score**	0.4609	0.8361	0.8562	**0.8811**	0.7928	0.7535
**MCC**	0.3884	0.8149	0.8359	**0.8651**	0.7667	0.724
**Kappa**	0.5185	0.3884	0.4236	**0.5292**	0.2212	0.1112
**Training Time(Sec)**	**24.284**	59.005	21.974	42.012	57.513	122.32

**Table 8:** Classification results at 100× magnification, after feature optimization on BreakHis dataset.

Performance Metric	Classifier					
	Tree	Narrow NN	Medium NN	Wide NN	Bi-Layer NN	Tri-Layer NN
**Accuracy**	**0.9962**	0.9913	0.9913	0.9904	0.9923	0.9759
**Error**	**0.0038**	0.0087	0.0087	0.0096	0.0077	0.0241
**Recall**	**0.9929**	0.9861	0.9886	0.984	0.9868	0.9545
**Specificity**	**0.9994**	0.9987	0.9987	0.9986	0.9989	0.9966
**Precision**	**0.9958**	0.9871	0.9864	0.9856	0.9892	0.9703
**False Positive Rate**	**0.00061**	0.0013	0.0013	0.0014	0.0011	0.0034
**F1_Score**	**0.9943**	0.9866	0.9873	0.9848	0.988	0.9605
**MCC**	**0.9938**	0.9853	0.9862	0.9834	0.9869	0.9583
**Kappa**	**0.9824**	0.9604	0.9604	0.956	0.9648	0.89
**Training Time(Sec)**	**10.465**	23.518	29.064	37.713	27.733	24.759

Tables 9 and 10 show the results of 200× magnification levels before and after feature optimization, respectively. WE can observe in Table 8 that Wide NN performed well than other classifiers due to their ability to detect features like texture, edges and shapes in images so they make precise selection of features, resulting improvement in precision and recall. In contrast, decision trees and narrow NNs often face limitations in data handling capacity, generalization, and scalability, leading to comparatively lower precision and recall. Whereas, after feature optimization, Trees have beaten in terms of whole performance metric to all classifiers, in comparison, due to decreased input complexity.

**Table 9:** Classification results at 200× magnification, before feature optimization on BreakHis dataset.

Performance Metric	Classifier					
	Tree	Narrow NN	Medium NN	Wide NN	Bi-Layer NN	Tri-Layer NN
**Accuracy**	0.5373	0.7851	0.8229	**0.8527**	0.7831	0.7682
**Error**	0.4627	0.2149	0.1771	**0.1473**	0.2169	0.2318
**Recall**	0.4444	0.7313	0.7704	**0.806**	0.718	0.6898
**Specificity**	0.9268	0.9666	0.9712	**0.9749**	0.9658	0.9637
**Precision**	0.4338	0.7186	0.7772	**0.8327**	0.715	0.6897
**False Positive Rate**	0.0732	0.0334	0.0288	**0.0251**	0.0342	0.0363
**F1_Score**	0.4378	0.7246	0.7733	**0.8185**	0.7147	0.6888
**MCC**	0.3648	0.691	0.7451	**0.7949**	0.6817	0.653
**Kappa**	0.5272	0.0175	0.1903	**0.3268**	0.0084	0.0565
**Training Time(Sec)**	**16.675**	89.393	27.628	46.028	113.82	122.35

**Table 10:** Classification results at 200× magnification, after feature optimization on BreakHis dataset.

Performance Metric	Classifier					
	Tree	Narrow NN	Medium NN	Wide NN	Bi-Layer NN	Tri-Layer NN
**Accuracy**	**0.995**	0.9881	0.9871	0.99	0.99	0.994
**Error**	**0.005**	0.0119	0.0129	0.01	0.01	0.006
**Recall**	**0.9905**	0.9842	0.982	0.9839	0.9845	0.9889
**Specificity**	**0.9992**	0.9984	0.9981	0.9986	0.9985	0.9991
**Precision**	**0.9947**	0.9798	0.9805	0.9851	0.9854	0.9926
**False Positive Rate**	**0.008**	0.0016	0.0019	0.0014	0.0015	0.001
**F1_Score**	**0.9926**	0.9819	0.9812	0.9845	0.9849	0.9907
**MCC**	**0.9918**	0.9802	0.9793	0.9831	0.9835	0.9899
**Kappa**	**0.9773**	0.9454	0.9409	0.9545	0.9545	0.9727
**Training Time(Sec)**	**8.916**	25.352	27.663	43.026	31.066	27.804

The meticulous examination of Tables 11 and 12 reveals interesting information. Before feature optimization, WideNN outshined all other classifiers due to more generalization ability than Tree, Narrow & Medium NN and it has less internal complexity than Bilayered and Trilayered NN. After feature optimization, the performance of Medium, Wide and Bilayered NN was equal in terms of accuracy but we can observe in Table 11 that Bilayered NN have more MCC, Kappa and F1_Score than Medium and Wide NN. So performance of Bilayered NN was best among all, in comparison. Although, Wide NN have more Precision (99.16%) than Wide NN (99.13%) but Medium NN have more F1_Score (99.08%) than Wide NN (98.96%) but equal Kappa and MCC, we can conclude that performance of Medium NN was better than Wide NN.

**Table 11:** Classification results at 400× magnification, before feature optimization on BreakHis dataset.

Performance Metric	Classifier					
	Tree	Narrow NN	Medium NN	Wide NN	Bi-Layer NN	Tri-Layer NN
**Accuracy**	0.5155	0.7467	0.8073	**0.8458**	0.7434	0.7137
**Error**	0.4845	0.2533	0.1927	**0.1542**	0.2566	0.2863
**Recall**	0.3985	0.6965	0.7578	**0.7931**	0.6759	0.6214
**Specificity**	0.9217	0.9599	0.9688	**0.9742**	0.9598	0.9552
**Precision**	0.4117	0.6919	0.7685	**0.8204**	0.6768	0.6399
**False Positive Rate**	0.0783	0.0401	0.0312	**0.0258**	0.0402	0.0448
**F1_Score**	0.4043	0.6919	0.7624	**0.8045**	0.675	0.627
**MCC**	0.3269	0.6531	0.732	**0.7813**	0.6357	0.5847
**Kappa**	0.5485	0.1364	0.1189	**0.2952**	0.1475	0.2361
**Training Time(Sec)**	37.505	53.866	**32.658**	49.44	174.69	186.32

**Table 12:** Classification results at 400× magnification, after feature optimization on BreakHis dataset.

Performance Metric	Classifier					
	Tree	Narrow NN	Medium NN	Wide NN	Bi-Layer NN	Tri-Layer NN
**Accuracy**	0.9901	0.9912	**0.9934**	**0.9934**	**0.9934**	0.9846
**Error**	0.0099	0.0088	**0.0066**	**0.0066**	**0.0066**	0.0154
**Recall**	0.9845	0.9877	0.9903	0.9878	**0.9922**	0.9724
**Specificity**	0.9985	0.9985	**0.999**	**0.999**	**0.999**	0.9976
**Precision**	0.9864	0.9897	0.9913	**0.9916**	0.9896	0.983
**False Positive Rate**	0.0015	0.0015	**0.001**	**0.001**	0.0018	0.0024
**F1_Score**	0.9853	0.9886	**0.9908**	0.9896	**0.9908**	0.9774
**MCC**	0.984	0.9872	0.9898	0.9887	**0.9899**	0.9753
**Kappa**	0.9547	0.9597	**0.9698**	**0.9698**	**0.9698**	0.9295
**Training Time(Sec)**	**13.398**	24.055	25.326	41.467	23.554	31.599

**Fig 4:**
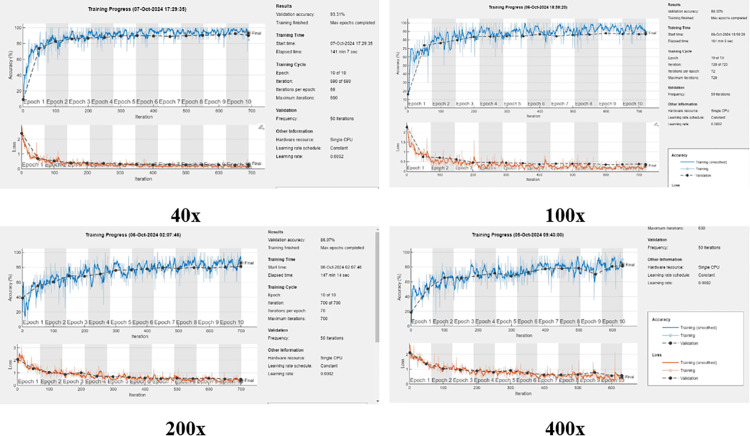
Training graphs of BreakHis dataset with respective magnification levels.

The training graphs illustrated in Fig 4 shows that proposed approach showed more convergence with increased accuracy at lower magnification level as and when magnification of dataset images increased, the convergence decreased to certain level and model gained accuracy after more iterations. Similarly, the error curve exhibits the converse. At lower magnification level of images, the error decreased after lesser iterations whereas, increase in magnification level, resulted reduction in error after greater number of iterations.

**Fig 5:**
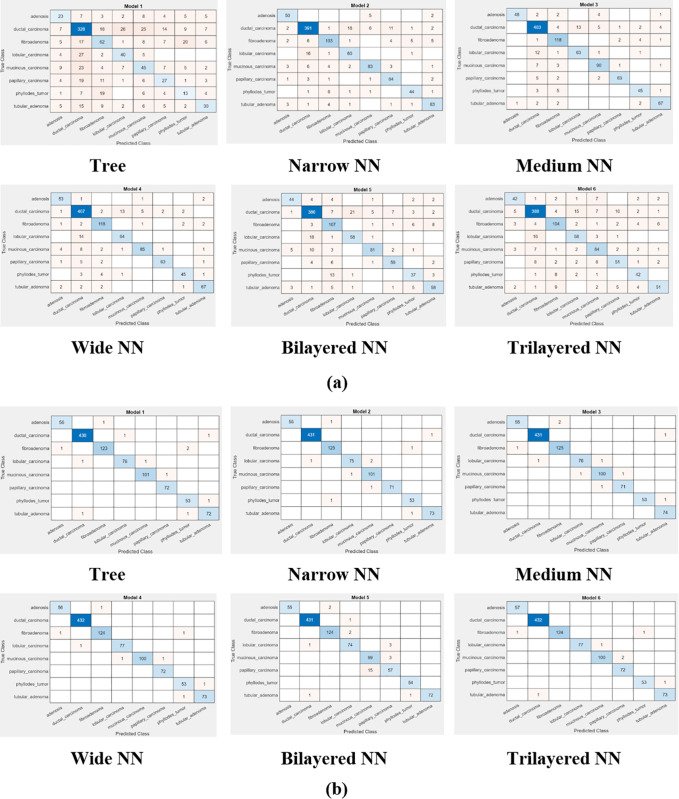
Confusion matrix of classifiers at 40× magnification (a) before optimization (b) after optimization.

**Fig 6:**
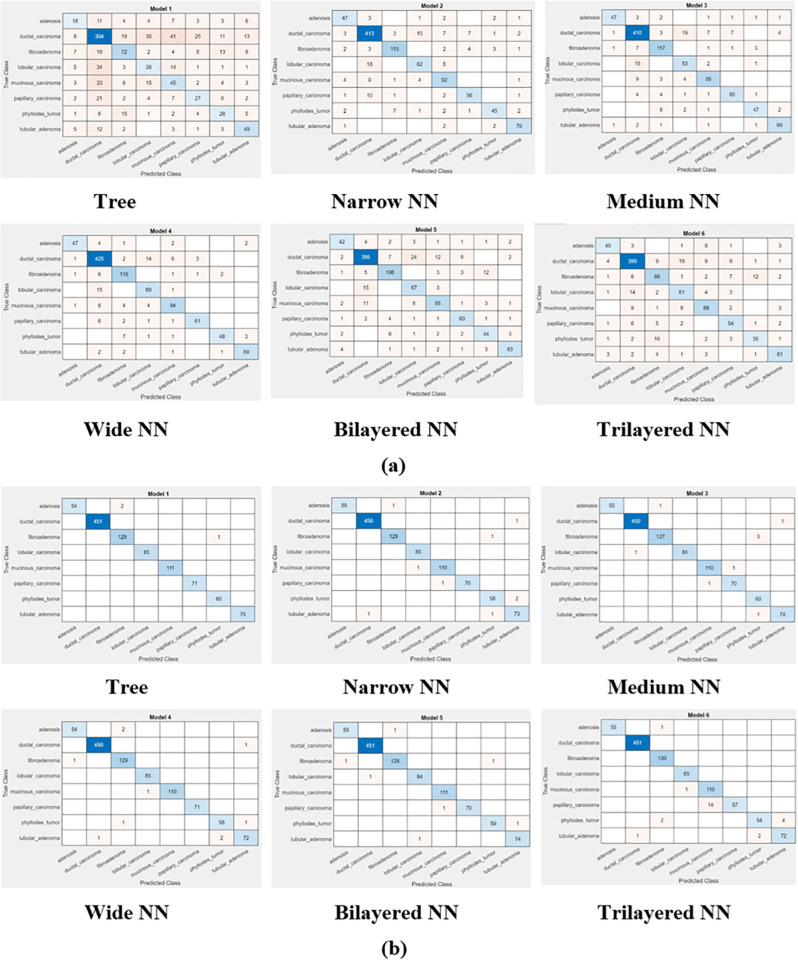
Confusion matrix of classifiers at 100× magnification (a) before optimization (b) after optimization.

Comparison of Figs 5 and 6 depicts that feature optimization has improved the classification accuracy, precision and recall. This, in turn, decreased the classification error, false negative rate and false positive rate. One can say that feature optimization improves the classification accuracy after selection of discriminant features from a given feature vector. The Wide NN performed well in terms of classification before feature optimization whereas after classification Wide NN and Trilayered NN equally performed better. Performance of Tree classifier was at lowest level of correct predictions, before optimization, due to increased input complexity. The reason behind improvement in classification results is selection of discriminant features by Flea Optimization Algorithm for classification.

**Fig 7:**
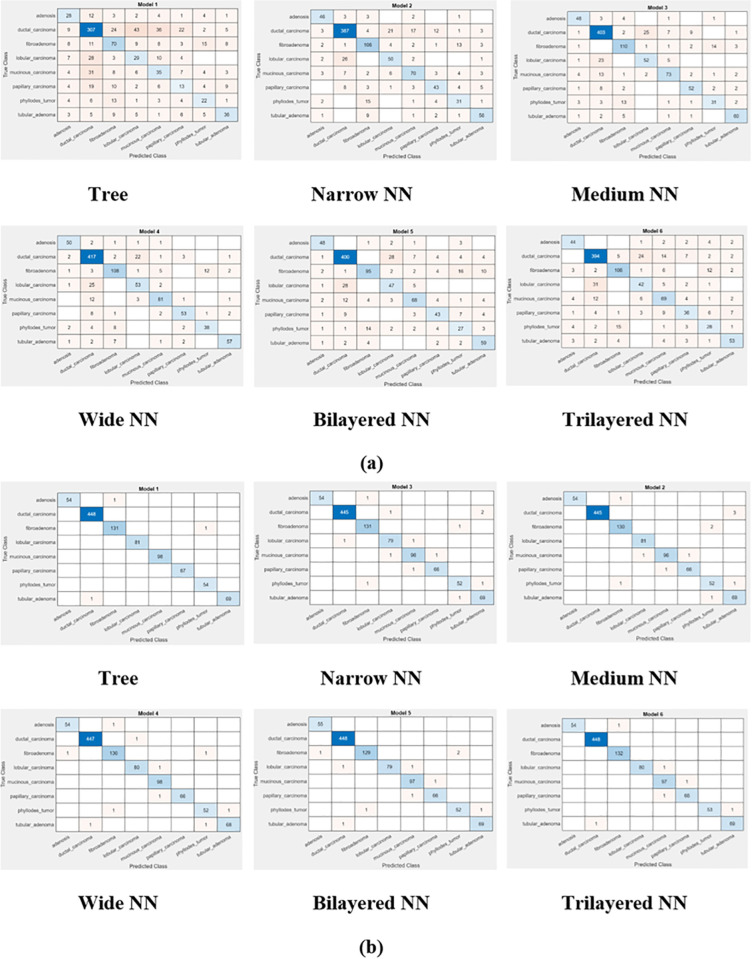
Confusion Matrix of classifiers at 200× magnification (a) before optimization (b) after optimization.

**Fig 8:**
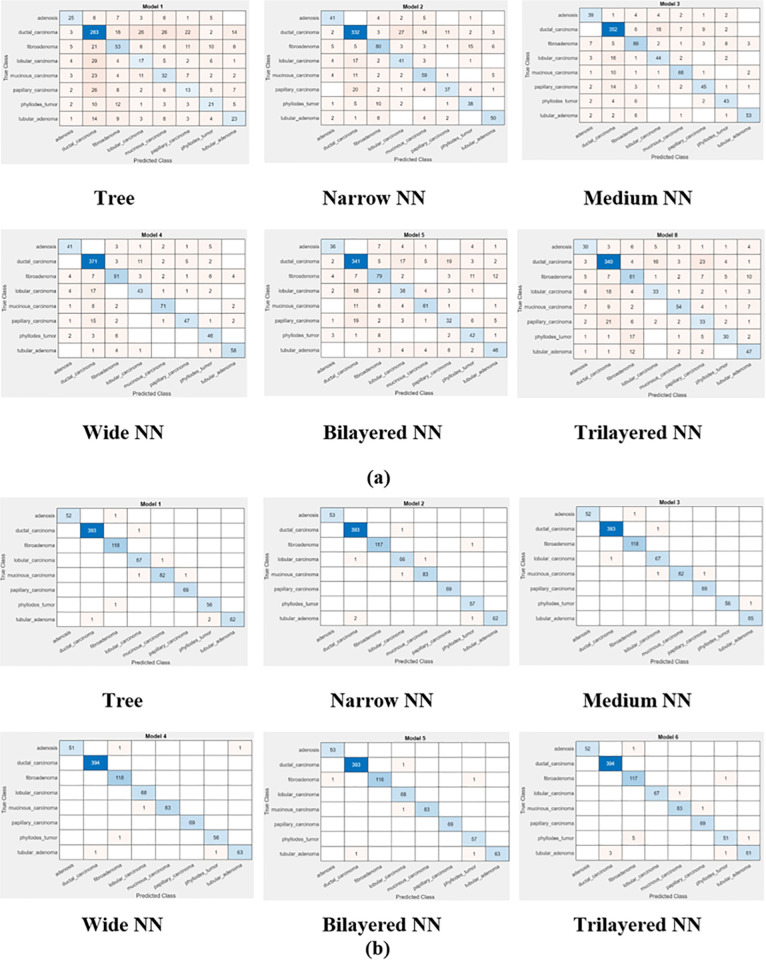
Confusion matrix of classifiers at 400× magnification (a) before optimization (b) after optimization.

We can observe a bit of performance degradation in Figs 7 and 8, before and after feature optimization, when magnification of the images has increased from 200× to 400×. The reason may be blurring of images which lacks in detection of edges, textures and shapes in images. This degradation affects the performance metric in a bad fashion. This, in turn, increases the classification error, false negative rate and false positive rate.

**Fig 9:**
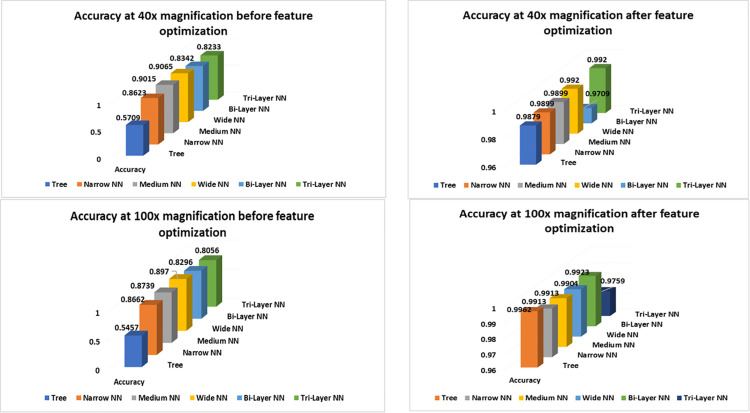
Accuracy graphs of classifiers at 40× and 100×.

As shown in Fig 9, classification accuracy increased up to 99.2% whereas, it was 90.65% before optimization in case of 40× magnification likewise, it increased from 89.7% to 99.23% in 100× scenario. The increase in accuracy is due to discriminatory abilities of selected features which help in detection of inter image dissimilarity. More the dissimilar images, more accurate classification will be and vice versa.

**Fig 10:**
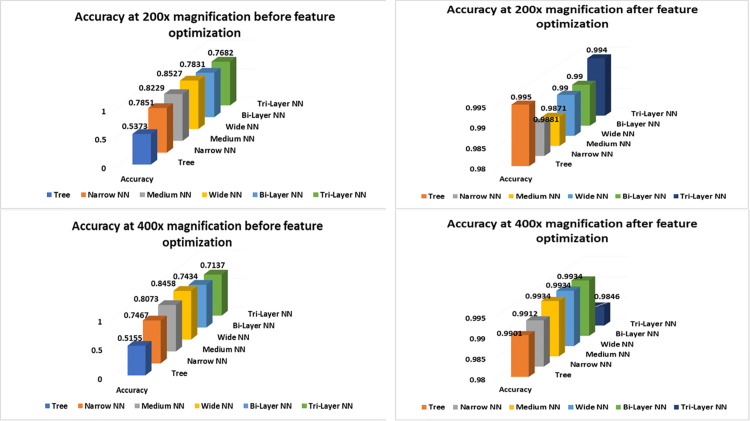
Accuracy graphs of classifiers at 200× and 400×.

**Fig 11:**
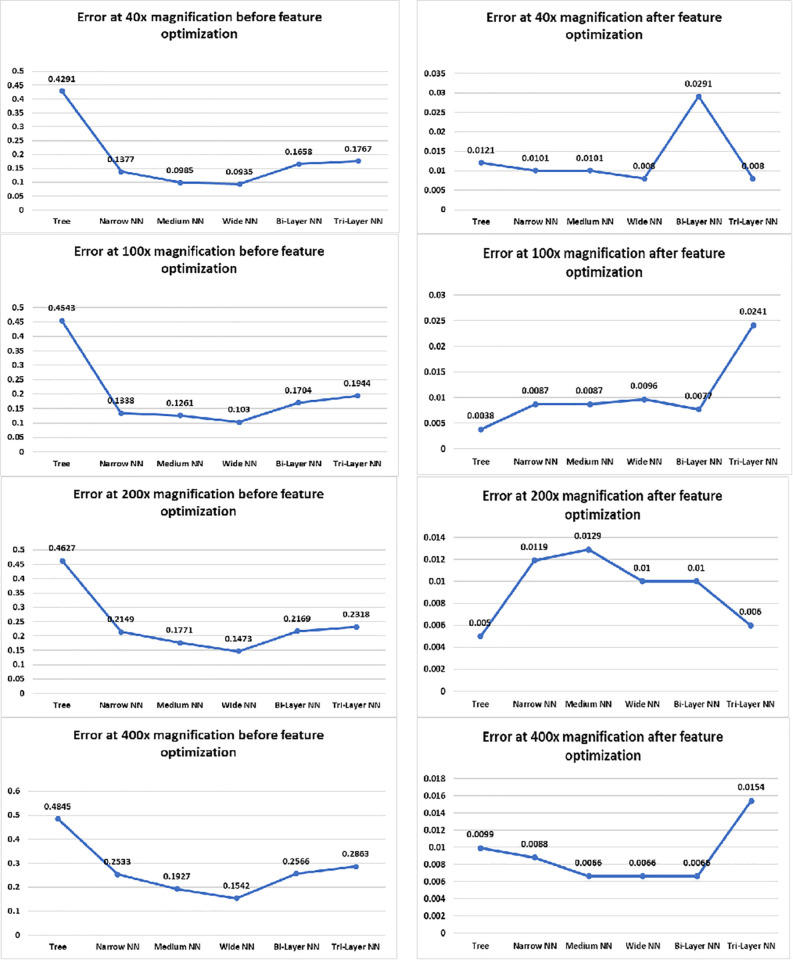
Error graphs of classifiers.

**Fig 12:**
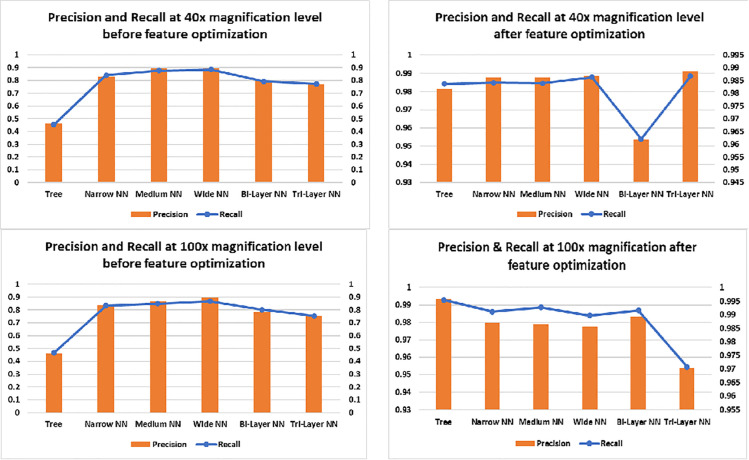
Precision and recall graphs of classifiers at 40× and 100×.

Accuracy graphs of 200× and 400× are listed in Fig 10 which show improvement in accuracy after FOA has been applied on the extracted features. Trees showed major improvement in both cases as their input complexity has been reduced and discriminant features helped them to find inter-image dissimilarity more accurately. In case of neural networks, Bilayered and Trilayered NN have performed well due to their property of detecting textures, shapes and edges in the image which make them superior from other neural networks. The extracted discriminant features help to find inter-image dissimilarity more accurately. Fig 11 shows the error percentage before and after feature optimization. It can be noticed from graphs that error was up to 42.90% and 45.43%, before feature optimization and after the feature optimization, the error has reduced to 0.8% and 0.38% respectively. Whereas, the minimum error before optimization was 9.35%. Maximum error reduction has been observed in Trees which is 45% and minimum in Wide NN which is 9% and the reason behind this phenomenon is classification accuracy before optimization. The accuracy of Trees was less so error rate was more, conversely, the Wide NN have More accuracy than Trees so their error rate was less. After application of FOA, classification accuracy of Trees was observed towards increase which, decreased the error rate. On the other hand, after feature optimization, there was comparatively small improvement in Wide NN (as it was performing a bit well before feature optimization) which caused less reduction in error. The graphs on the right side of Fig 12 shows the precision and recall after feature optimization. It is clear from the graph that minimum 95.38% (Bilayered) and maximum 99% (Trilayered NN) precision has been obtained by neural networks, after feature optimization due to their inherent property of detecting features like texture, edges and shapes in images so they make precise selection of features, resulting improvement in precision and recall. On the other hand, for neural networks, it was minimum 78% (Trilayered NN) and maximum 90% (Wide NN) before feature optimization. Moreover, the high dimensional data is handled efficiently by Trilayered and Bilayered NN by using regularization techniques such as batch normalization, dropout and weight regularization which causes improved precision and recall. In contrast, decision trees and narrow NNs often face limitations in capacity, generalization, and scalability, leading to comparatively lower precision and recall before feature optimization but when the input of Trees and Narrow NN was optimized set of features, the precision and recall increased due to decrease in input complexity. It shows that feature optimization not only increases the accuracy but it also increases the precision and recall. Consequently, error, false positive rate and training time reduces as well.

**Fig 13:**
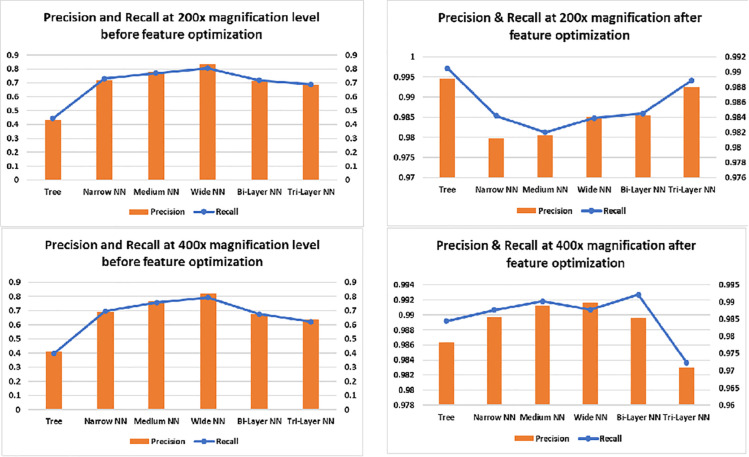
Precision and recall graphs of classifiers at 200× and 400×.

**Fig 14:**
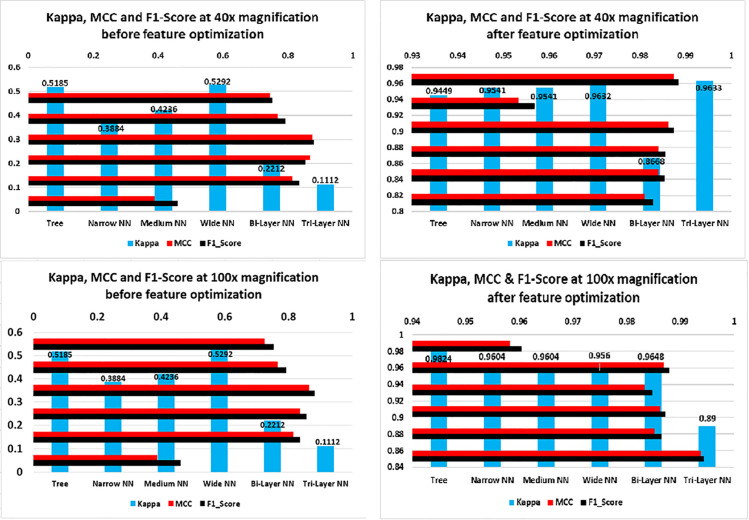
Kappa, MCC and F1_Score graphs of classifiers at 40× and 100×.

**Fig 15:**
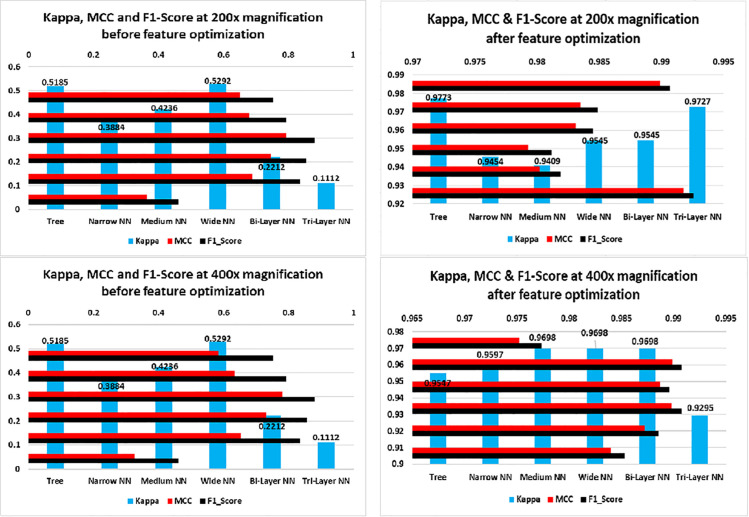
Kappa, MCC and F1_Score graphs of classifiers at 200× and 400×.

Fig 13 illustrates the results for 200× and 400× magnification levels. The left side graphs show the performance before optimization therein one can infer that maximum recall was about 80%. Whereas, after feature optimization, recall approaches approximately 99% and precision up to 99%. The increase in recall may be due to more elaborated and zoomed in features of the images which helped in efficient classification of images By considering all true positives, true negatives, false positives and false negatives, MCC provides good insights of the model. We can see in Fig 14 that before feature optimization the values of MCC were smaller but after feature optimization, the model’s MCC score improved. One can see the minimum score achieved after feature optimization is 95.5%(40× magnification) and maximum is 98.99% (100× magnification). Likewise, Kappa and F1_Score have improved due to increased precision and recall. As F1_Score is harmonic mean of precision and recall, an increase in precision and recall also give a rise to F1_Score. Kappa indicates that model has accurately predicted instances to their correct labels. Increase in all three parameters means that model has performed well on most of the instances. We can see in Fig 15 that at 400× magnification level, the MCC, Kappa and F-1 Score all decreased than 200× magnification level in both left and right graphs this is due to two reason; firstly, before optimization, at input there were too much features and due this dimensionality curse, the classifiers could not classify the images with more accuracy which result decrease in precision and recall. The decrease in precision and recall cause a decrease in MCC, Kappa and F1_Score. Secondly, after optimization, the classifiers may not be able to detect texture, shape or edges due to magnification of the images which resulted extraction of less discriminant features; as a result model’s precision and recall decreased, which in turn, decreased the MCC, Kappa and F-1 Score of the model.

**Fig 16:**
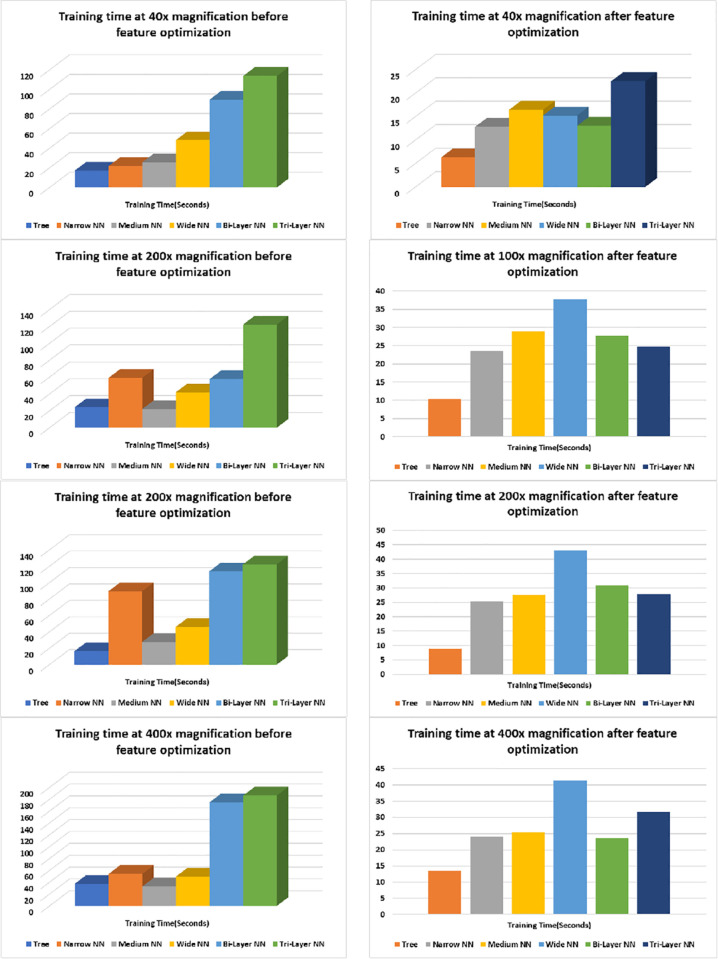
Training time graphs of classifiers.

Although training time has not much contribution in improvement of accuracy rather it helps to complete the training in shorter time interval and reduces the time complexity of the algorithm. The graphs on the left in Fig 16 show the training time before feature optimization and the graphs on right side show training time after feature optimization. We can see that the reduction of features has decreased timed complexity approximately 50% for the Tree, Narrow NN and Medium NN whereas, it has decreased approximately 85% in case of Bilayered and Trilayered NN. This reduction in training time enables the proposed approach to complete in comparatively less finite time with more accuracy, precision, recall, MCC, Kappa and F-1 Score and reduced error, and false positive rate.

**Fig 17:**
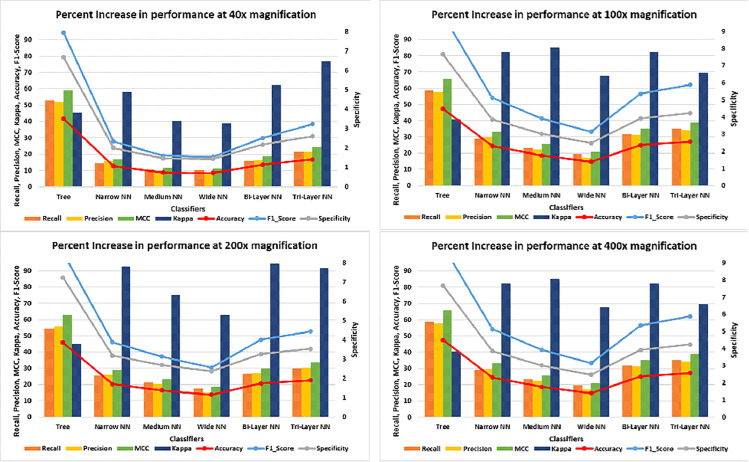
Percent improvement in error, recall, specificity, precision and accuracy.

The graphs in Fig 17 show detailed analysis of improvements, made by the proposed approach in the performance metric. We can see that in case of tree, on average 55% recall, precision and 40% Kappa has increased which resulted increased accuracy whereas MCC, accuracy, F-1 Score and specificity got rise on average 35%, 32%, 90% and 80%, respectively which shows that models has predicted most of the instances correctly. We can see that Kappa has increased for all classifiers; i.e. minimum 40% (Medium & Wide NN) and maximum about 90% (Trilayered NN). The increase in Kappa denotes that model has predicted well for most of the instances against their labels during classification, after feature optimization. The F-1 score and accuracy curves depicts that after feature optimization, model has improved the accuracy, precision and recall for all the classifiers.

It can be observed from the Table 13 that proposed approach has performed well than state-of-the-art techniques. We have obtained greater accuracy in less computational and time complexity in all magnification levels of BreakHis dataset. A statistical t-test was conducted on the learning results and found a value of 0.712 as t-statistics with 5 degrees of freedom. The corresponding two-tailed p-value is observed to be 0.51, that exceeded the conventional significance threshold of 0.05. It is also worth to mention that 95% confidence interval for the mean accuracy ranged from approximately 0.986 to 0.996, further supporting the conclusion that the model’s accuracy reliably aligns with the expected benchmark.

**Table 13:** Comparison of results with state-of-the-art approaches.

Methodology	Accuracy (%)	Dataset Used
DenseNet [25]	98.13%	BreakHis
VGG [26]	89%	BreakHis
CNN with LSTM [27]	91%	BreakHis
Primal-Dual Multi-Instance SVM [28]	89.8%	BreakHis
Single task CNN, multi-task CNN [29]	83.33%	BreakHis
Proposed Method	99.20% at 40× magnification	BreakHis
	99.62% at 100× magnification	
	99.50% at 200× magnification	
	99.34% at 400× magnification	

### Ablation study

**Fig 18:**
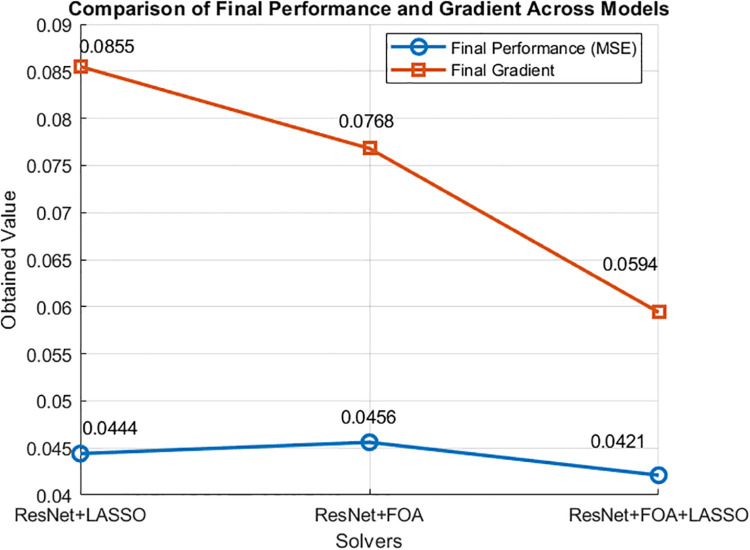
Performance comparison of ResNet-LASSO, ResNet-FOA and hybrid ResNet-FOA-LASSO.

The performance of LASSO and FOA has been evaluated by using three ResNet-based feature selection scenarios (LASSO, FOA, and hybrid ResNet-FOA-LASSO) in terms of convergence, computational efficiency and predictive accuracy. We took Mean Squared Error (MSE) as performance indicator, lower value of MSE will indicate better accuracy. ResNet-LASSO required 308 epochs to reach the stopping criterion, it took 94 seconds as training time, and achieved 0.0444 MSE score with a gradient of 0.0855. The performance of ResNet-FOA was a bit better as it took 312 epochs to attain MSE score of 0.0768 and used 47 seconds as training time. The hybrid ResNet-FOA-LASSO approach outperformed both methods, converging in only 205 epochs, consumed 17 seconds as training time and provided lowest MSE 0.0421 with a gradient value of 0.0594. These results demonstrate that ResNet-FOA-LASSO is an efficient and accurate method for optimizing ResNet-extracted features, achieving lower network loss in fewer epochs and with less computational complexity. Experimental results are listed in Table 14. Fig 18 shows the performance comparison of the scenarios.

**Table 14:** Comparison of ResNet-based feature selection methods in terms of training epochs, time, final performance (MSE), and gradient. Lower MSE and gradient indicate better accuracy and convergence.

Model	Epochs	Time	MSE	Gradient
ResNet + LASSO	0 → 308	0:01:34	0.0444	0.0855
ResNet + FOA	0 → 312	0:00:47	0.0456	0.0768
ResNet + FOA + LASSO	0 → 205	0:00:17	0.0421	0.0594

### Inter-image dissimilarity

Inter-image dissimilarity is a numerical measure which is used to measure degree of differences in images of a dataset. This measure has been used to examine the structural separability of breast cancer tissue subtypes in the feature space (which has been generated by the proposed approach). After extraction of deep features using a pretrained ResNet, an optimal subset of highly discriminative features was selected by using FOA and LASSO. Pairwise distances between all images was computed using the Euclidean distance measure. For each class, intra-class dissimilarity was used as the mean distance among samples belonging to the same subclass, whereas inter-class dissimilarity was measured as the mean distance between samples of a given subclass and all samples of the remaining classes. The difference between these two metrics, helped to identify the class margin and characterized the compactness and separability of each class in the learned feature space. This measure gives a quantitative basis that how well the feature-extraction stage differentiates visually similar tissue patterns before classification. Table 15 shows intra and inter class dissimilarity along with Class Margin and Accuracy.

**Table 15:** Intra-class, inter-class dissimilarity, class margin, and classification accuracy for breast cancer classification.

Class	Intra	Inter	Margin	Accuracy
Adenosis	18.13	41.939	23.809	0.8018
Ductal carcinoma	2.43	11.703	9.28	0.5930
Fibroadenoma	5.32	17.74	12.42	0.7554
Lobular carcinoma	0.23	8.93	8.70	1.0000
Mucinous carcinoma	0.24	9.56	9.33	0.9470
Papillary carcinoma	0.22	9.66	9.45	1.0000
Phyllodes tumor	1.09	10.97	9.89	0.9956
Tubular adenoma	0.95	9.49	8.53	1.0000

The observed dissimilarity structure in extracted features shows the inherent variability (in shape, texture, structure and appearance of breast cancer tissues) has been closely aligned with the classification performance. Classes such as papillary carcinoma, lobular carcinoma, phyllodes tumor, and tubular adenoma exhibited very low intra-class dissimilarity (≤1.0) and relatively high inter-class dissimilarity (≈9–11) which resulted large positive class margins and achieved good accuracy (99–100%). Whereas, adenosis and ductal carcinoma demonstrated higher intra-class dissimilarity (18.13 and 2.43, respectively), indicating significant intra-class heterogeneity, as a result, classification accuracy decreased to 80% and 59%, respectively. A strong and consistent relationship has been observed between class margin and classification accuracy which indicates that greater separability in the dissimilarity space results more reliable predictions. These findings validate that the deep features learned by ResNet inherently encode inter-image dissimilarity patterns. Moreover, the classification performance is determined by how the images are distributed in terms of their dissimilarity in a given dataset.

## Section IV: Conclusions

Based upon the comprehensive simulations and discussion on the graphs, tables and findings of the proposed framework, the following conclusions can be drawn:

- The proposed deep neural network architecture consists of 146 layers. As a result of this optimization in deep neural network, the number of learnable parameters decreased from 23.7M to 16.8M. Consequently, time complexity decreased.
- A novel Flea Optimization Algorithm has been introduced in the study for selection of discriminant features, from a given feature set. Optimized feature vector improves accuracy and reduces time complexity.
- At input stage, pre-processing was performed on dataset through augmentation techniques such as shear, reflection and rotation to enhance model’s robustness. As a result, model became robust and accuracy of model improved which resulted improvement in performance metric.
- On average Wide NN performed well, before and after feature optimization. The time complexity is also acceptable in both scenarios.
- It has been observed when the magnification level of images is increased then convergence becomes slower. The convergence at 40× magnification was faster as compared to 100× and convergence at 100× was faster as compared to 200× and so on. All the magnification levels of BreakHis dataset have been considered and an accuracy of 99.20% at 40× magnification, 99.62% at 100× magnification, 99.50% at 200× magnification and 99.34% at 400× magnification has been achieved. To evaluate the effectiveness of the proposed approach, we compared proposed approach with DenseNet, VGG, LSTM, Primal-Dual Multi-Instance SVM, Single task CNN and multi-task CNN considering accuracy, precision, recall, F-1 measure, Kappa, computational, time, and space complexity. Moreover, the proposed approach outperformed all the deep learning models in comparison.
- The performance of the proposed approach is compared with DenseNet, VGG, LSTM, Primal-Dual Multi-Instance SVM, Single task CNN and multi-task CNN using following performance metric: i)accuracy; ii) F-1 measure; iii) Recall; iv) Precision; v. Error; vi) KAPPA; and vii) MCC. After experiments, results showed that proposed approach outperformed all approaches, in comparison. The improved performance metric of the proposed model makes it ideal for physicians to use it in real life breast cancer diagnosis.
- An ablation study has been conducted to evaluate the effectiveness of FOA and LASSO with ResNet architecture and concluded that alone LASSO or FOA underperformed but the combination of both (hybrid approach) has resulted an increase in classification accuracy.
- The MCC values observed from 0.9534 to 0.9873 and Kappa values were from 0.8668 to 0.9633. Moreover, t-test gave a value of 0.712 as t-statistics with 5 degrees of freedom. The corresponding two-tailed p-value is observed to be 0.51, that exceeded the conventional significance threshold of 0.05 which gave us 95% confidence interval.

In future, one can exploit Flea Optimization Algorithm for hyperparameter tuning of deep learning architecture. The implementation of these architectures can be performed on microchips to have practical implementation in the health sciences and other allied domain.
